# Prevalence and Associated Factors of Eating Disorders Among Undergraduate Students at the University of Kirkuk, Iraq: A Cross-Sectional Study

**DOI:** 10.7759/cureus.64247

**Published:** 2024-07-10

**Authors:** Nashwan N Hasan, Bestoon M Ahmed, Omed H Mehammed-Ameen, Mariwan Q Hama Rash

**Affiliations:** 1 Community Health, Kirkuk University College of Nursing, Kirkuk, IRQ; 2 Maternal and Neonatal Health, Kirkuk University College of Nursing, Kirkuk, IRQ; 3 Nursing, Al-Kitab University, Kirkuk, IRQ

**Keywords:** mental health, feeding behavior, diet, students, eating disorders

## Abstract

Background

Eating disorders (EDs) are significant health hazards among university students because of various stressors and lifestyle changes. Therefore, in this study, we examine the prevalence of EDs among undergraduate students at the University of Kirkuk and explore the relationships between EDs and sociodemographic factors.

Methods

The research employed a cross-sectional design and took place from June 3, 2023, to April 30, 2024. The Eating Attitudes Test-26 (EAT-26), a standardized survey, was used to collect data on participants’ eating attitudes and sociodemographic information. The data were analyzed using descriptive statistics and inferential statistical tests.

Results

The overall sample comprised 513 individuals aged 18 to 30 years. The findings indicated that 25.7% of the participants were prone to developing EDs. The results revealed significant differences in ED scores between those who engaged in self-starvation for weight loss, daily weight monitoring, or experienced weight-based bullying (p < 0.001). Moreover, this study found that BMI has a positive correlation with the dieting and oral control subscales (p < 0.05), and it also has a positive correlation with the total EAT-26 score (p < 0.01). Interestingly, the dieting subscale negatively correlates with age, while the EAT-26 total score negatively correlates with both students’ academic stage and satisfaction with their field of study.

Conclusions

Based on the EAT-26 assessment, a quarter of the students were at risk of developing EDs. The data also show that BMI is positively associated with the risk of EDs. These findings highlight the need for targeted mental health interventions and support systems within universities.

## Introduction

Eating attitude is an internationally recognized term that includes beliefs, thoughts, feelings, and food-related behaviors that influence an individual's eating behavior [1]. Eating disorders (EDs), defined by the American Psychological Association as "a persistent disturbance of eating or eating-related behavior that results in the altered consumption or absorption of food and that significantly impairs physical health or psychosocial functioning" [2], can indicate individuals' subconscious thoughts and body image and their emotional evaluation of their own and others' bodies. Studies indicate that underlying attitudes toward food play a fundamental role in shaping dietary preferences, encompassing both nutritious and less nutritious choices, as well as eating behaviors. These choices and behaviors subsequently influence hunger levels, cravings, and the propensity to develop EDs. Therefore, these choices and behaviors significantly influence hunger levels, cravings, and the tendency to develop EDs [3]. The psychological aspects of eating attitudes are significant relative to general health and well-being, hence the need for a balanced eating lifestyle [1]. The results of this disorder may be extremely complex, with severe physical complications such as suicide and death; this is particularly true for anorexia nervosa [4].

In recent decades, rapid and large-scale industrialization and urbanization have led to substantial increases in EDs globally [5], such as in the Asian-Pacific regions. EDs are common, affecting up to 5% of the general population [2]. Owing to varying regions and cultural contexts, the prevalence of EDs among undergraduate students undergoing their education varies significantly, ranging from 13.97% [6] to 40.9% [7]. However, the rates in Western Asia are slightly higher compared to globally reported cases. Students in Arab countries also have a mean prevalence of 31.4% of EDs, with higher rates among females and essential risk factors such as age, gender, BMI, and eating patterns [8]. This underscores a call for further detailed research in developing countries [9]. A recent study at Al-Qadisiyah University, located in the south of Iraq, documented that 21.84% of undergraduate students were positive for ED risk factors, further confirming this need [10].

Despite growing awareness, the prevalence of EDs among undergraduate students in developing regions such as Iraq remains under-researched. Therefore, in this study, we aim to determine the prevalence of EDs among undergraduate students at the University of Kirkuk. Additionally, the study will explore the relationship between EDs and various factors, aiming to fill knowledge gaps and provide data to inform targeted interventions and support strategies.

## Materials and methods

Design of the study

This research used a cross-sectional design.

Setting and duration of the study

The research was conducted at the University of Kirkuk from June 3, 2023, until April 30, 2024.

Tools of the study

All participants received a comprehensive explanation of the study's objectives, methods, potential drawbacks, benefits, and the confidentiality of their responses before completing the questionnaire. The participants were notified of their entitlement to either participate or decline involvement in the research.

Part I of the tool collects sociodemographic data such as gender, age, BMI, satisfaction with the study's major, and weight-related behaviors. Part II utilized the Eating Attitudes Test-26 (EAT-26), a widely recognized and validated self-report questionnaire. The study employed both English and Arabic versions of the EAT-26 after obtaining permission from the original authors [1,11].

The EAT-26 questionnaire scores its items on a six-point Likert scale and divides them into three subscales: (1) dieting, (2) bulimia and food preoccupation, and (3) oral control. The scoring system allocates three points for "always," two points for "usually," one point for "often," and zero points for "sometimes," "rarely," and "never." By summing the scores of all items (1-26), the total score can range from 0 (minimum score) to 78 (maximum score). The EAT-26 questionnaire uses a scoring system with higher scores indicating a greater likelihood of disordered eating attitudes. Notably, Garner et al. found that a score of 20 or higher on the EAT-26 was indicative of the "clinical range." This means that people scoring 20 or higher may benefit from further evaluation by a qualified healthcare professional to check for a possible ED diagnosis [11].

Sampling and sample

The G*Power program was used to determine the minimum sample size required to achieve adequate statistical power for our study. The analysis revealed that a minimum sample size of 343 subjects was required. This conclusion was based on several key parameters: a small effect size of 0.2, which is common in social science research when small differences or relationships are expected in the data; a 95% confidence level, which ensures that the results are statistically significant and the probability of error is only 5%; and a 5% margin of error, a standard for ensuring the accuracy of survey estimates [12].

An online questionnaire via Google Forms (Google, Inc., Mountain View, CA) was created and shared as a link with college and department administrators to be sent to students, utilizing convenience sampling. A total of 513 responses from 18 different faculties were collected, forming the sample for this research. Each student was limited to completing the survey only once. Participation was voluntary, and obtaining the respondent's permission was mandatory to proceed.

Participants had to be currently enrolled as university students at the University of Kirkuk, aged 18 to 30, and provide informed consent. Students outside this age range or those who did not complete the study questions were excluded.

Ethical consideration

Before conducting the research (No. 2, 2/10/2023), the Ethical Committee of the University of Kirkuk, College of Nursing, granted approval. Participants' permission before data collection was granted.

Statistical analysis

The data were entered and converted into a digital format using Microsoft Excel and then analyzed using the Statistical Product and Service Solutions (SPSS, version 27; IBM SPSS Statistics for Windows, Armonk, NY). Summary statistics, including frequency and percentage, were used to describe sociodemographic variables and EAT-26 scores.

Because of the non-normal distribution of the data, nonparametric statistical techniques were employed. Spearman's correlation was used to investigate the associations between variables. Additionally, the study utilized the Mann-Whitney U test to compare means. The statistical analyses were performed using a significance level of p < 0.05.

## Results

A study of 513 undergraduate students aged 18-30 reported that over half had a BMI within the healthy range, and 41.3% were satisfied with their majors (Table 1). Approximately 2.5% reported using supplements, and 16.8% starved themselves because of dieting. Approximately 18.1% engaged in daily weight monitoring and experienced weight-based bullying.

**Table 1:** Undergraduate students demographic and behavioral characteristics (n = 513). SD: standard deviation

Variables	Frequency	Percentage
Age (Years)		
18-19 years old	44	8.6
20-21 years old	225	43.9
22-23 years old	159	31.0
24-25 years old	51	9.9
>25 years old	34	6.6
Mean (SD) 22.05 (± 2.95)		
Gender		
Male	193	37.6
Female	320	62.4
BMI		
Underweight	45	8.8
Normal	293	57.1
Overweight	137	26.7
Obese	38	7.4
Satisfaction with the major of studying		
Very unsatisfied	63	12.3
Unsatisfied	55	10.7
Neutral	88	17.2
Satisfied	212	41.3
Very satisfied	95	18.5
Used weight loss supplements over the last month		
No	500	97.5
Yes	13	2.5
Last month experiencing severe hunger to decrease body weight		
No	427	83.2
Yes	86	16.8
Daily weighting		
No	420	81.9
Yes	93	18.1
Being bullied because of weight		
No	420	81.9
Yes	93	18.1

**Figure 1:**
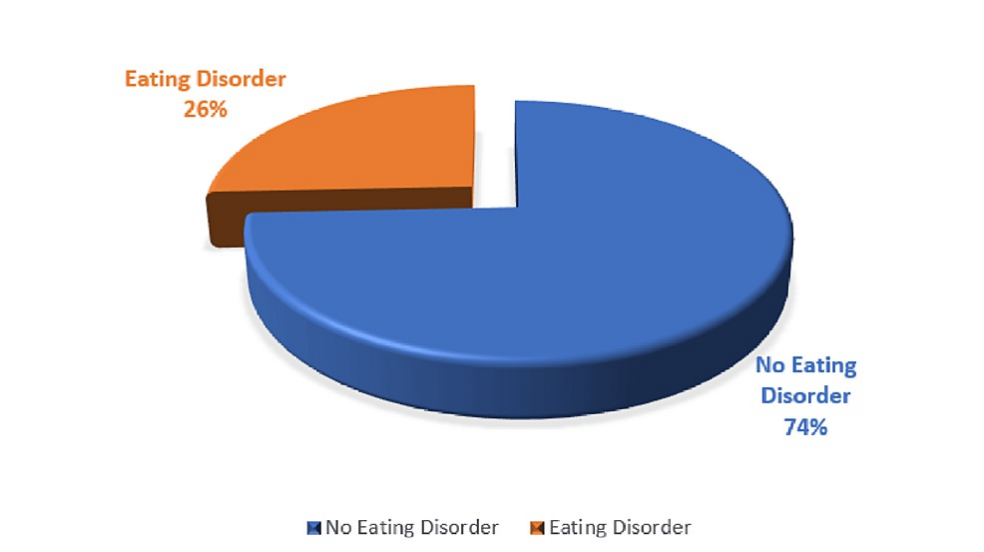
Prevalence of eating disorders among undergraduate students. %: percentage

The EAT-26 assessment revealed that 25.7% of participants were at risk of developing EDs, while 74.3% did not meet the criteria for an ED (Figure 1).

The Mann-Whitney U test in Table 2 indicates that gender did not have a significant relationship with ED scores. However, compared to those who did not, there were significant differences between ED scores and students who used diet pills in the past month (p = 0.002). Similarly, significant differences were observed in ED scores for those who engaged in self-starvation for weight loss, daily weight monitoring, or experienced weight-based bullying (p < 0.001).

**Table 2:** Mann-Whitney U test to compare sample sociodemographic characteristics and eating disorder indicators. U: Mann-Whitney U test statistic

Categories	Group	Frequency	Mean Rank	Sum of Ranks	U	P value
Gender	Male	193	270.62	52,229.50	28251.500	0.106
	Female	320	248.79	79,611.50		
Used weight loss supplements over the duration of the last month.	No	500	253.68	126,840.50	1,590.500	0.002
	Yes	13	384.65	5,000.50		
Last month experiencing severe hunger to decrease body weight.	No	427	233.11	99,537.50	8,159.500	< 0.001
	Yes	86	375.62	32,303.50		
Daily weighting	No	420	245.77	103,224.00	14,814.000	< 0.001
	Yes	93	307.71	28,617.00		
Being bullied because of weight	No	420	245.77	103,224.00	14,814.000	< 0.001
	Yes	93	307.71	28,617.00		

In Table 3, Spearman's correlation test reveals some intriguing correlations between factors and EDs. BMI has a positive correlation with the dieting and oral control subscales (p < 0.05), and it also shows a positive correlation with the total EAT-26 score (p < 0.01). Interestingly, the dieting subscale negatively correlates with age. Moreover, the EAT-26 total score negatively correlates with both academic stage and satisfaction with the field of study. Additionally, the study found positive correlations between the dieting subscale and both the bulimia and oral control subscales, as well as a positive correlation between bulimia and oral control themselves.

**Table 3:** Spearman's correlations between the eating disorder scale and subscales, age, BMI, stage of study, and satisfaction with their major. The results are statistically significant at the 0.01 level** and at the 0.05 level*. Spearman's rho: Spearman's rank correlation coefficient; EAT-26: Eating Attitudes Test-26

Spearman's Rho	1	2	3	4	5	6	7
1- Age							
2- BMI	0.099*						
3- Stage	0.446**	-0.026					
4- Field of study satisfaction	0.007	0.014	0.034				
5- Dieting	-0.102*	0.105^*^	-0.042	0.015			
6- Bulimia	-0.015	0.052	-0.068	-0.024	0.223^**^		
7- Oral control	0.050	0.105^*^	0.041	-0.023	0.176^** ^	0.254^**^	
8- EAT-26	0.045	0.183^**^	-0.114^* ^	-0.104^*^	-0.015	-0.036	-0.065

## Discussion

According to the EAT-26 assessment, 25.7% of the students in the study were at risk of developing EDs. This prevalence rate is consistent with some previous research; however, it differs considerably from others, which may imply the possibility of cultural and regional differences in ED risks. For example, studies done in the United Arab Emirates and Malaysia revealed prevalence rates of 13.97% and 14.9%, respectively [6,13].

However, a study among Pakistani students reported a significantly higher prevalence of 40.9%, indicating that the likelihood of developing EDs is culturally dependent [7]. Similarly, the current study found a risk prevalence of EDs in line with US college students reported as high as 28%, indicating a similar occurrence of EDs across diverse academic settings [14]. This highlights the universal nature of EDs among student populations and underscores the need for targeted interventions. Additional factors, such as the unstable security situation and uncertain future in Iraq, may contribute to the prevalence of EDs [15].

Research findings from multiple studies provide insights into the relationship between EDs and gender. Some studies show differences in specific behaviors between genders, such as girls being more preoccupied with body image and boys engaging in extreme dieting behaviors [16,17]. Overall, there seems to be no significant difference in the prevalence of EDs between genders [18]. These findings suggest that while there may be gender-specific nuances in certain eating behaviors, the overall relationship between EDs and gender is not statistically significant. Students who reported taking diet pills to lose weight in the past thirty days had significantly higher ED scores. Diet pill use is considered an extreme weight-control practice [19]. Hazzard et al. found a significant association between diet pill use and weight control. Those who took diet pills were more likely to receive a first-time ED diagnosis within the next five years compared to those who did not use them. This is worrying behavior, as it indicates a greater emphasis on quick weight loss than on healthy habits [20].

Students who had starved themselves to lose weight in the previous month had significantly higher ED scores. Other research findings also support the idea that starvation for weight loss is associated with significantly higher scores for EDs [21,22].

Daily weighing was also significantly associated with higher ED scores. This behavior signals a concern with body weight and shape that is typical of eating disorders, notably anorexia nervosa and bulimia nervosa. These behaviors are characteristic of EDs such as anorexia nervosa, where patients experience extreme weight loss and an intense fear of gaining it, or bulimia nervosa, characterized by binge eating, followed by compensatory measures such as vomiting or overexercising [23].

Weight-bullying experiences were significantly associated. This discovery emphasizes the role of social processes, such as peer victimization, in ED development. Weight teasing may be considered a form of bullying and has been associated with body image dissatisfaction and self-esteem, both key risk factors for EDs [23]. Furthermore, studies have shown that body-related bullying triggers a desire for weight loss in young individuals, who are more prone to negative health behaviors such as skipping meals, smoking, and alcohol consumption [24].

Spearman rank correlations indicated statistically significant associations between different sociodemographic variables and EDs among the University of Kirkuk undergraduate students. This analysis focuses on the interaction of patterns of disordered eating behavior and other variables; among them are age, BMI, academic stage, and satisfaction with the field of study.

The negative correlation between age and dieting behaviors suggests that as students grow older, they are less likely to engage in dieting behaviors. Older individuals may have developed more stable eating habits or have a better understanding of their nutritional needs, leading to a decreased reliance on restrictive dieting practices [25].

The relationship between BMI and EDs is multifaceted, as evidenced by various correlations. A positive correlation between BMI and the total EAT-26 score suggests that a higher BMI is associated with a higher risk of EDs, possibly due to body dissatisfaction [26]. Moreover, the positive correlation between BMI and oral control and diet scores implies that individuals with a higher BMI may exhibit more control over their eating, potentially as a compensatory behavior [27].

Students who reported higher levels of satisfaction with their branch of study exhibited lower total scores on the EAT-26 assessment, indicating a lower risk of EDs. This suggests that satisfaction with one's academic branch may contribute to better overall mental health and reduced stress, thereby decreasing the likelihood of engaging in disordered eating behaviors. This highlights the importance of control found to mediate the association between need satisfaction and ED pathology [28]. To address this, fostering a more positive attitude toward mental health in Iraqi communities is essential. Improving education, raising awareness, and increasing access to mental health services and supervision can achieve this [29].

This study's cross-sectional design, limited to a single year, restricts its ability to capture long-term influences on eating behaviors. Longitudinal studies could provide a clearer picture. Additionally, relying solely on the EAT-26 questionnaire might not yield the most accurate prevalence estimates. Including clinical diagnoses alongside the EAT-26 could improve accuracy. These limitations highlight areas for future research to refine our understanding of ED risk factors among Iraqi university students.

## Conclusions

This study highlights a significant prevalence of EDs among undergraduate students at the University of Kirkuk, with 25.7% at risk based on the EAT-26 assessment. The findings indicate that diet pills, starvation, daily weight monitoring, and weight-based bullying experiences are all significantly associated with higher ED scores. The data also show that older students and those more advanced in their studies tend to engage less in dieting behaviors, while a higher BMI correlates with increased ED risks. Additionally, greater satisfaction with the student's field of study is associated with lower ED scores.
